# Supplementary material for: A genome-wide meta-analysis uncovers six sequence variants conferring risk of vertigo
Source: Commun Biol. 2021 Oct 7;4:1148. doi: 10.1038/s42003-021-02673-2 (PMC8497462; doi:10.1038/s42003-021-02673-2)
Supplement: Supplementary file 2 — Supplementary information [file 42003_2021_2673_MOESM2_ESM.pdf]

Supplementary information

A genome-wide meta-analysis uncovers six sequence variants conferring risk of vertigo

Astros Th Skuladottir, Gyda Bjornsdottir, Muhammad Sulaman Nawaz, Hannes Petersen, Solvi Rognvaldsson, Kristjan Helgi Swerford Moore, Pall I Olafsson, Sigurður H Magnusson, Anna Bjornsdottir, Olafur A Sveinsson, Gudrun R Sigurdardottir, Saedis Saevarsdottir, Erna V Ivarsdottir, Lilja Stefansdottir, Bjarni Gunnarsson, Joseph B Muhlestein, Kirk U Knowlton, David A Jones, Lincoln D Nadauld, Annette M Hartmann, Dan Rujescu, Michael Strupp, G Bragi Walters, Thorgeir E Thorgeirsson, Ingileif Jonsdottir, Hilma Holm, Gudmar Thorleifsson, Daniel F Gudbjartsson, Patrick Sulem, Hreinn Stefansson, and Kari Stefansson

Contents

Supplementary Figure 1. Evidence of colocalization 2
Supplementary Figure 2. Manhattan plot for individual datasets 3
Supplementary Figure 3. Q-Q plots. 4
Supplementary Figure 4. Genetic correlation analysis. 5
Supplementary Figure 5. Gene-based genome-wide association analysis of vertigo. 6
Supplementary Figure 6. Risk comparison of the most common subtypes of vertigo. 7
Supplementary Figure 7. The first 20 principal components in the UK. 8
Supplementary Figure 8. The first 20 principal components in the US. 9
Supplementary Table 1. Heterogeneity and Random effects model 10
Supplementary Table 2. Gene-based genome-wide association analysis of vertigo. 11
Supplementary Table 3. ARHI and hearing loss association 12
Supplementary Table 4. Translation of codes in the UK dataset 13
Supplementary References 14

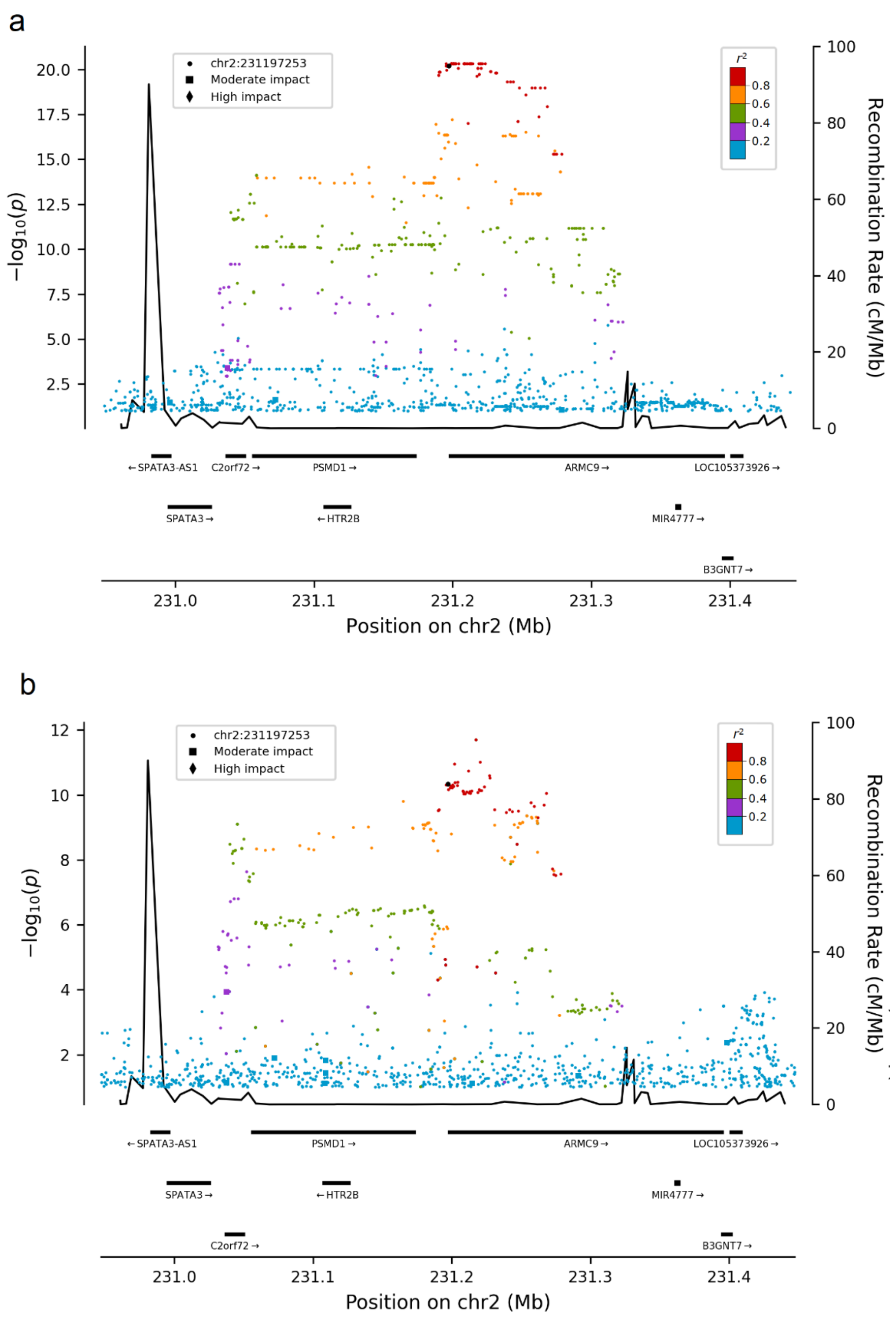

Supplementary Figure 1. Evidence of colocalization

Association results at the *ARMC9* locus show a high probability of a shared variant for a) adipose eQTL and b) vertigo. Variants are colored by the degree of correlation (r^2) with the lead variant, which is colored black. Functional variants have a moderate impact (square) or a high impact (diamond). The -log$_{10}$*P*-values on the left y-axis (two-sided logistic regression) are plotted for each variant against their chromosomal position (x-axis). The right y-axis shows calculated recombination rates based on the Icelandic data at the chromosomal location, plotted as solid black lines.

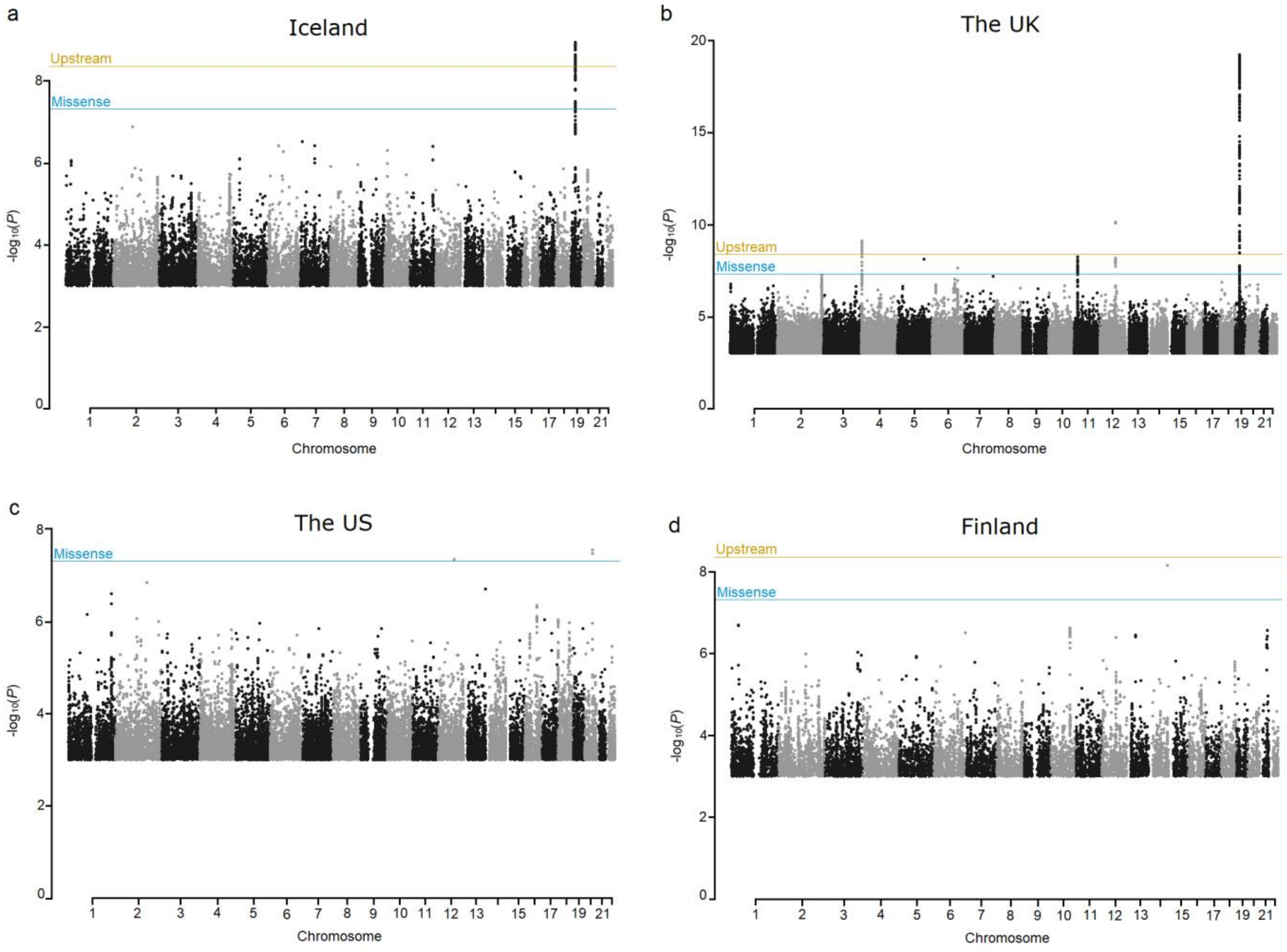

Supplementary Figure 2. Manhattan plot for individual datasets

a) Iceland (deCODE genetics), b) the UK (UK Biobank), c) the US (Intermountain), and d) Finland (Finngen). The -$\log_{10}P$-values (y-axis) are plotted for each variant against their chromosomal position (x-axis). The horizontal lines represent the adjusted variant-class threshold (blue for missense variants [$P \leq 4.9 \times 10^{-8}$] and orange for upstream variants [$P \leq 4.4 \times 10^{-9}$]).

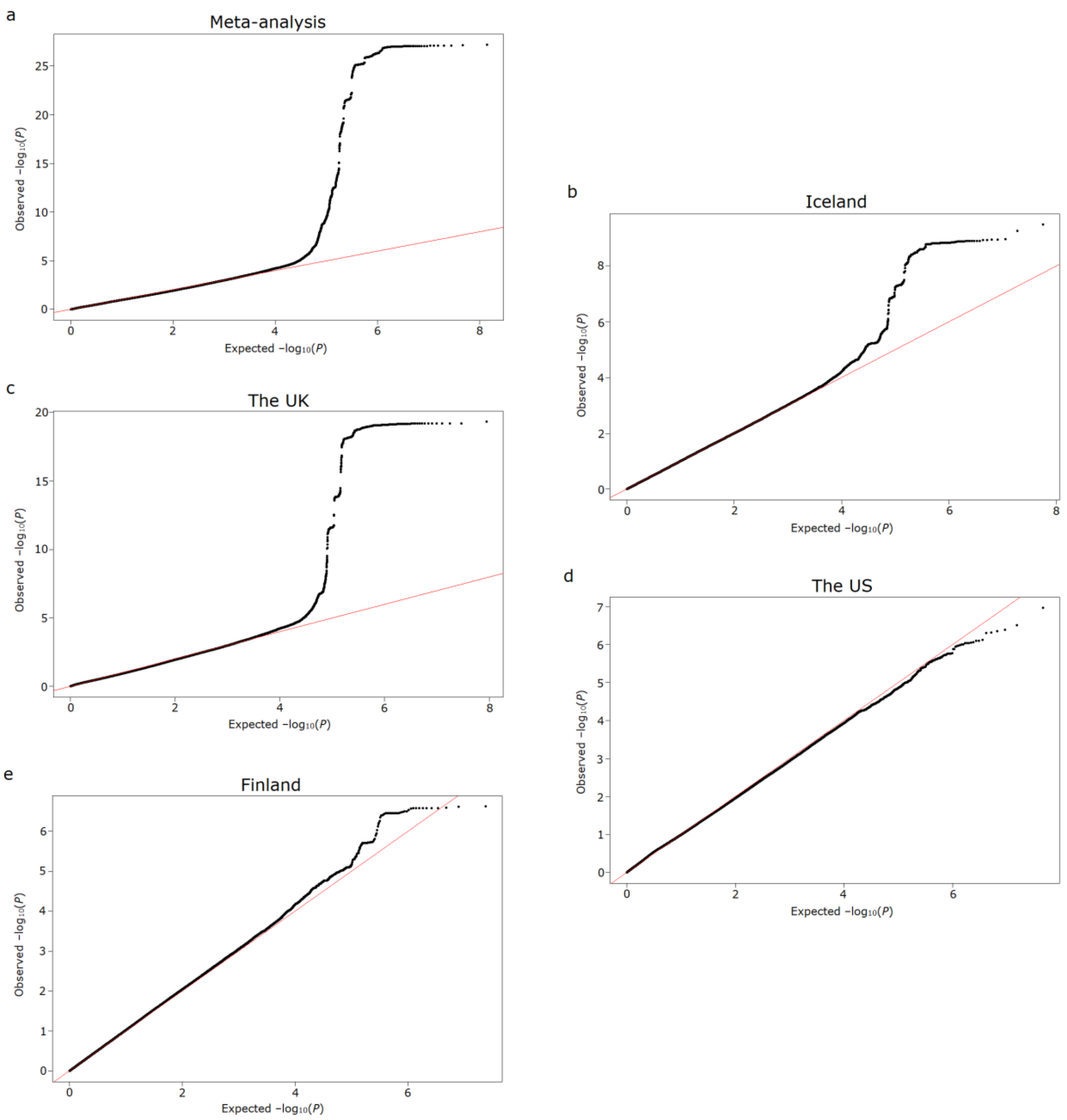

Supplementary Figure 3. Q-Q plots.
Q-Q plots of the *P*-values for sequence variants from the a) meta-analysis, and the GWASs from b) Iceland, c) the UK, and d) the US, and e) the summary statistics from Finland. The observed -log$_{10}$*P*-values (y-axis) are plotted for each variant with imputation information above 0.8 and MAF > 0.01% against the theoretical distribution (red line, x-axis). There is no systematic genomic inflation where the values follow the theoretical distribution. The values that deviate from the theoretical distribution indicate the sequence variants that associate significantly with vertigo.

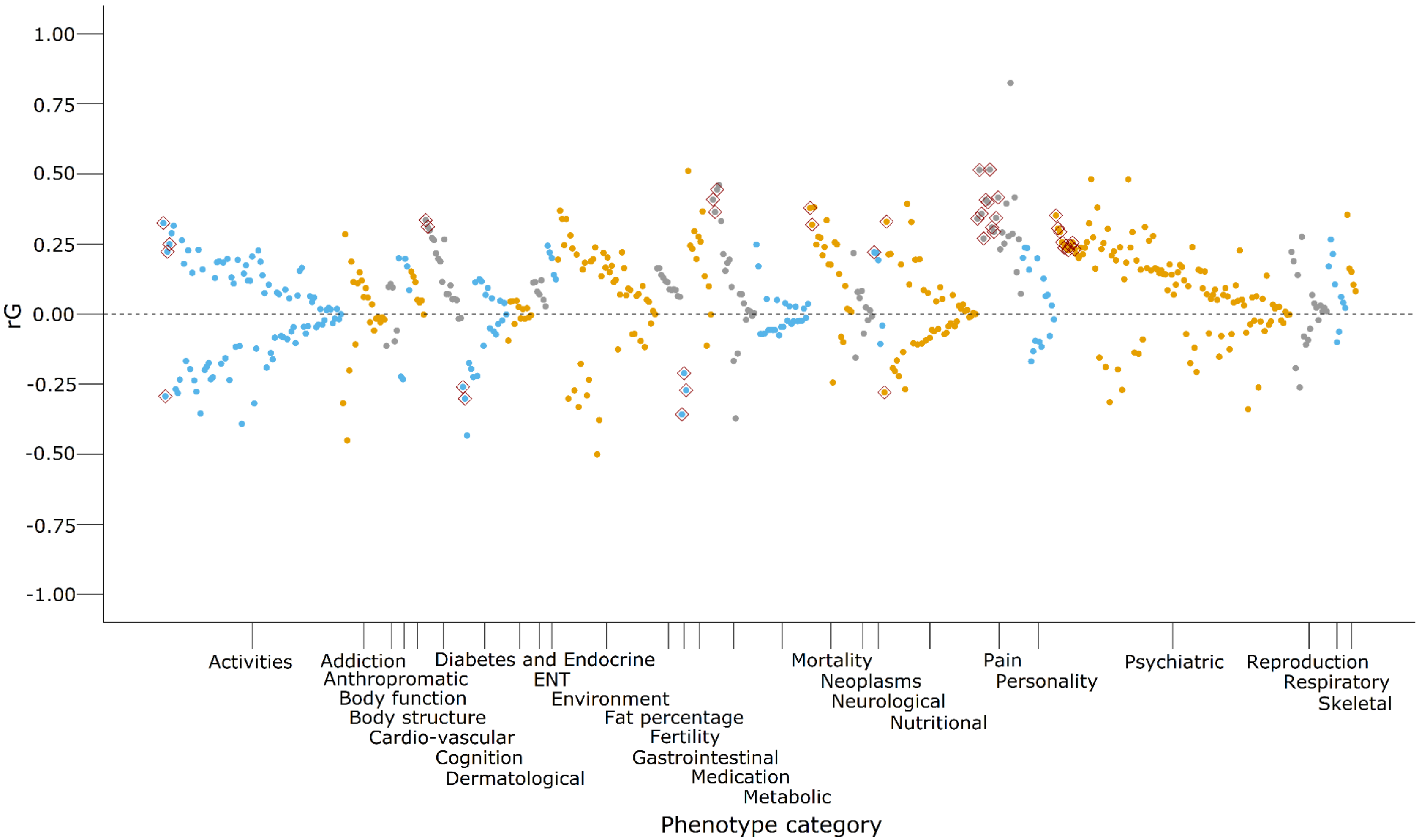

Supplementary Figure 4. Genetic correlation analysis.

Genetic correlation between the vertigo meta-analysis and previously published GWASs. The y-axis shows the genotypic correlation factor (rG) and the x-axis shows a phenotype category. A significant genetic correlation is noted by a red diamond around the data point. The colors of the data points (blue, orange, and gray) define each phenotype category and distinguish it from the next phenotype category.

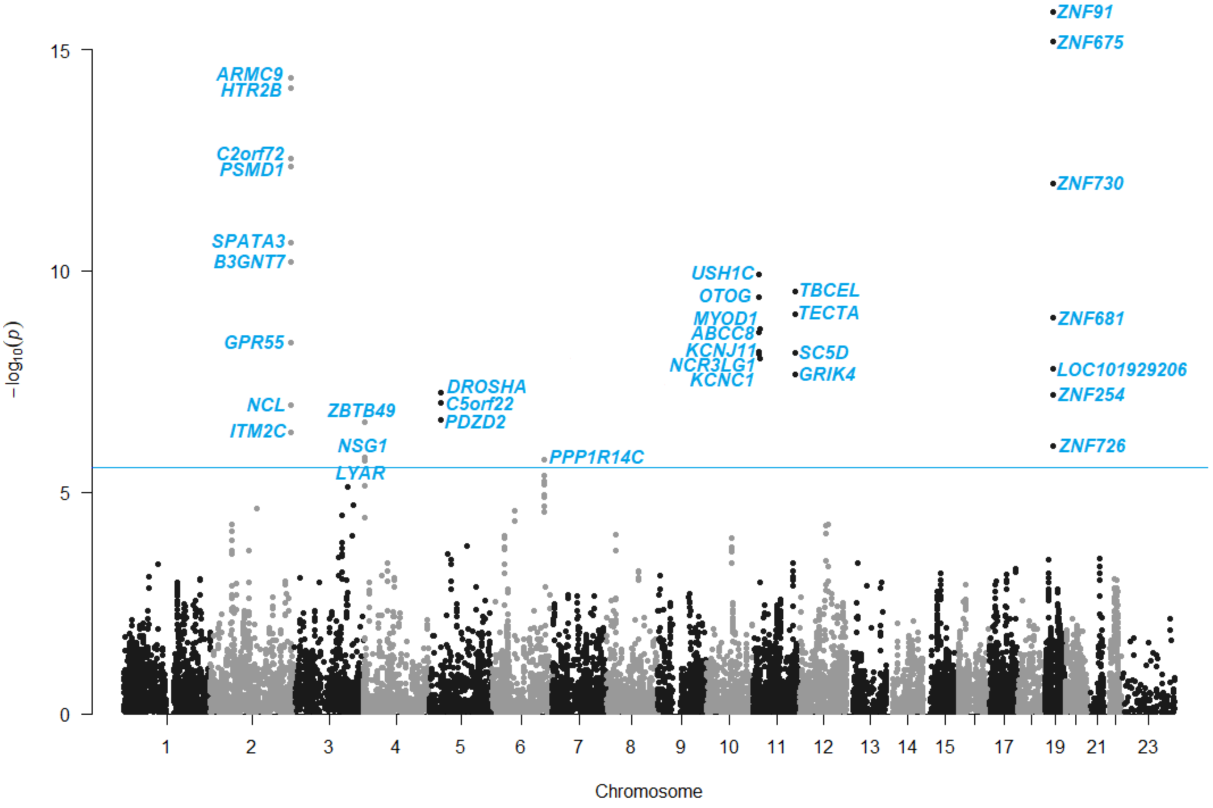

Supplementary Figure 5. Gene-based genome-wide association analysis of vertigo. The genes identified are marked in blue. The horizontal line indicates the significance threshold ($P \leq 2.7 \times 10^{-6}$).

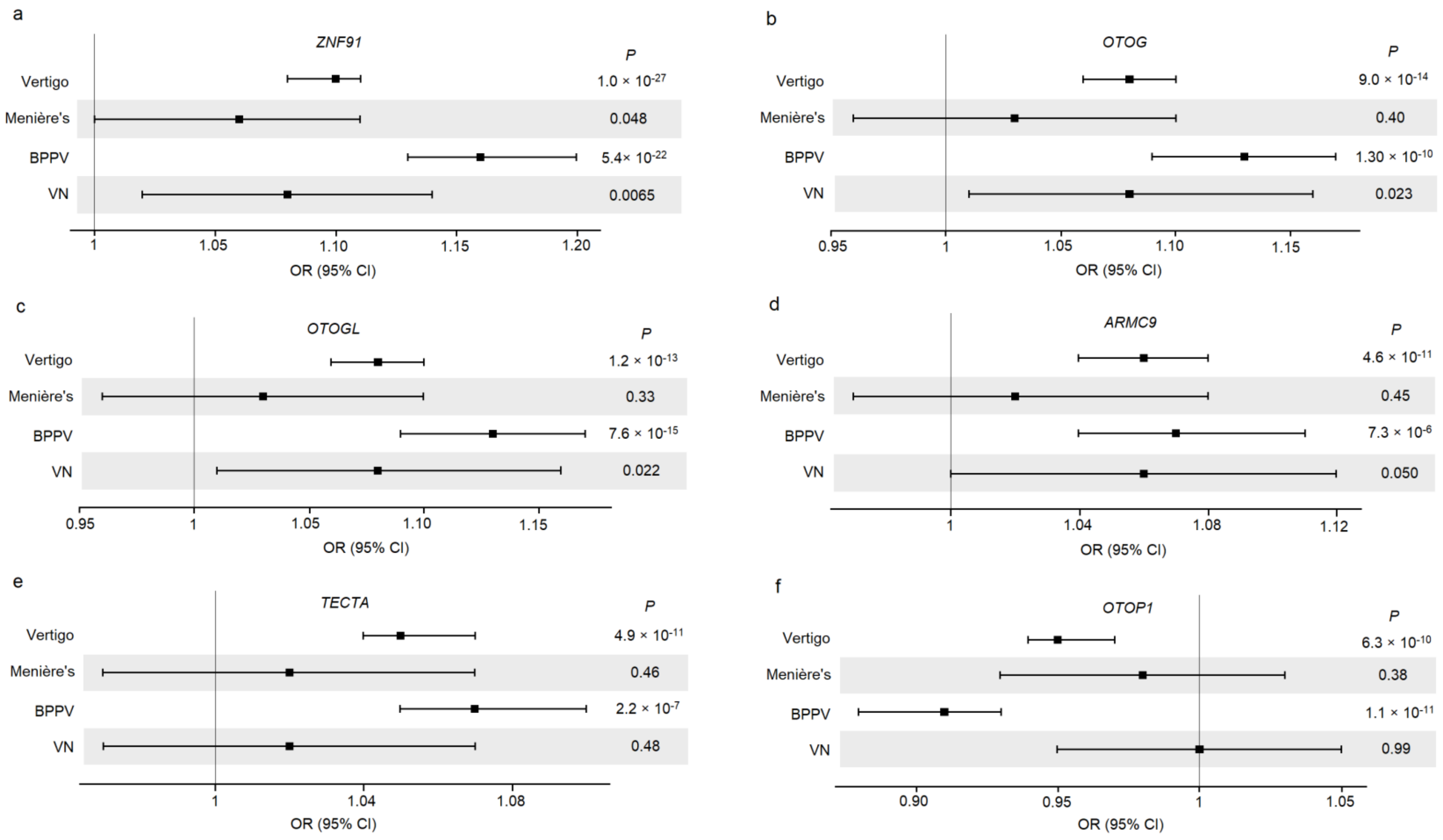

Supplementary Figure 6. Risk comparison of the most common subtypes of vertigo.
The significant risk alleles associating with vertigo at a) *ZNF91*, b) *OTOG*, c) *OTOGL*, d) *ARMC9*, e) *TECTA*, and f) *OTOP1*. The subtypes are classified using ICD-10 code H81.0 (Ménière‘s disease), H81.1 (BPPV), H81.2 (vestibular neuritis [VN]). For comparison, the risk of each allele is shown for the vertigo meta-analysis. The error bars indicate 95% CI.

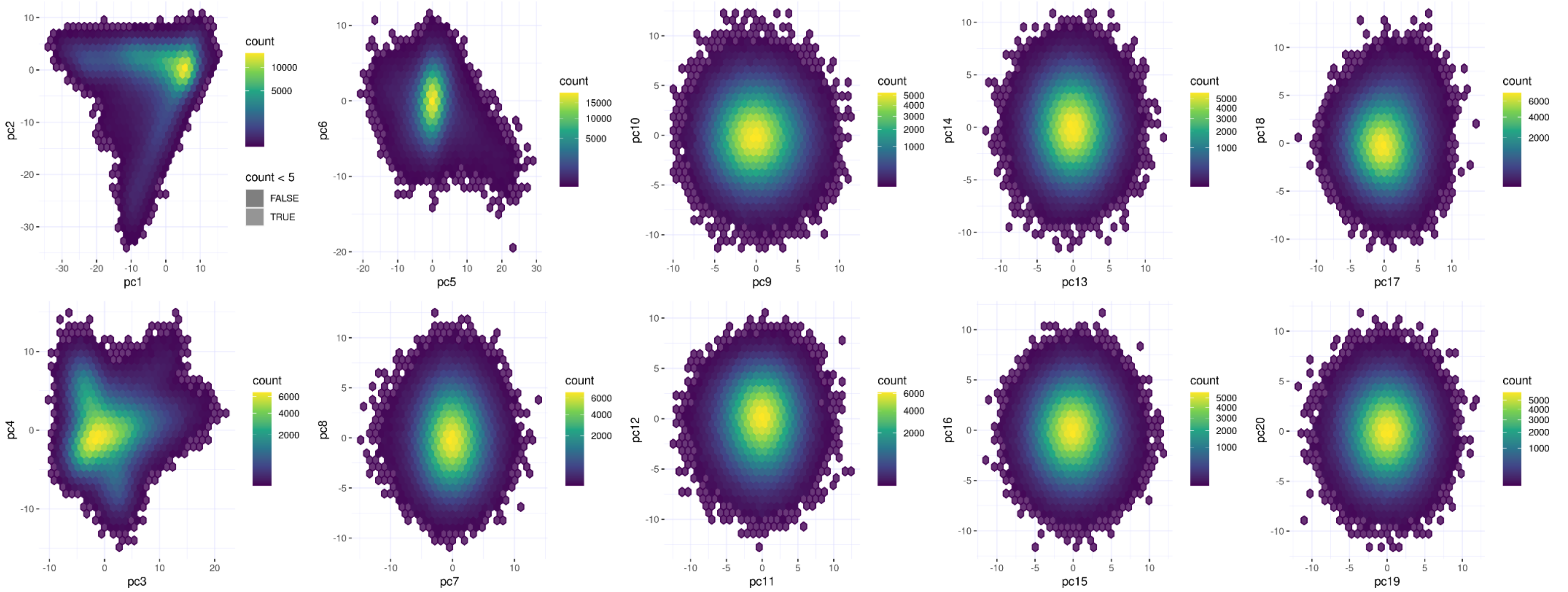

Supplementary Figure 7. The first 20 principal components in the UK.
The color bar indicates how many datapoints are in each hexagon. The hexagons containing fewer than 5 datapoints are more transparent (represented by light gray, indicating transparency) than the ones with more than 5 datapoints (represented by dark gray, indicating color concentration).

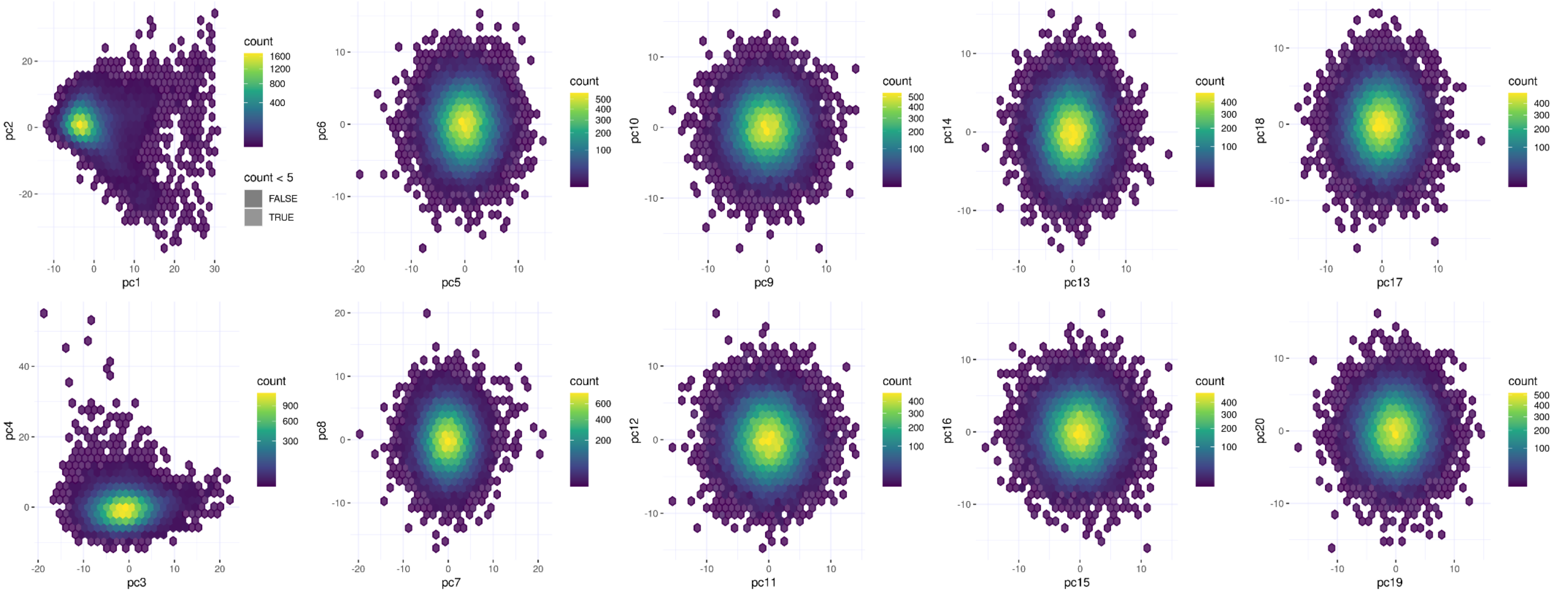

Supplementary Figure 8. The first 20 principal components in the US.
The color bar indicates how many datapoints are in each hexagon. The hexagons containing fewer than 5 datapoints are more transparent (represented by light gray, indicating transparency) than the ones with more than 5 datapoints (represented by dark gray, indicating color concentration).

Supplementary Table 1. Heterogeneity and Random effects model
P-het P-value for test of heterogeneity in the effect estimates between Iceland, the UK, the US, and Finland.

Risk allele	LD class size (r^2 > 0.8)	Gene	*P*-het	*P* random effect
rs428549-G	123	*ZNF91*	3.4×10^{-4}	2.0×10^{-26}
rs7130190-T	5	*OTOG*	0.098	4.1×10^{-13}
rs10862089-T	4	*OTOGL*	0.0040	1.1×10^{-12}
rs6753393-C	51	*ARMC9*	0.085	1.3×10^{-10}
rs612969-G	0	*TECTA*	0.015	1.1×10^{-10}
rs2272744-C	72	*OTOP1*	0.032	7.2×10^{-10}

Supplementary Table 2. Gene-based genome-wide association analysis of vertigo. The genes and loci associating with vertigo. The significance threshold is 2.7×10^{-6} based 18,815 genes tested.

Ensembl ID	Gene	Locus	*P*
ENSG00000167232	*ZNF91*	19p12	1.4×10^{-16}
ENSG00000197372	*ZNF675*	19p12	6.4×10^{-16}
ENSG00000135931	*ARMC9*	2q37.1	4.4×10^{-15}
ENSG00000135914	*HTR2B*	2q37.1	7.5×10^{-15}
ENSG00000204128	*C2orf72*	2q37.1	2.9×10^{-13}
ENSG00000173692	*PSMD1*	2q37.1	4.4×10^{-13}
ENSG00000183850	*ZNF730*	19p12	1.1×10^{-12}
ENSG00000173699	*SPATA3*	2q37.1	2.2×10^{-11}
ENSG00000156966	*B3GNT7*	2q37.1	6.3×10^{-11}
ENSG00000006611	*USH1C*	11p15.1	1.2×10^{-10}
ENSG00000154114	*TBCEL*	11q23.3	2.9×10^{-10}
ENSG00000188162	*OTOG*	11p15.1	3.9×10^{-10}
ENSG00000109927	*TECTA*	11q23.3	9.7×10^{-10}
ENSG00000196172	*ZNF681*	19p12	1.1×10^{-9}
ENSG00000129152	*MYOD1*	11p15.1	2.1×10^{-9}
ENSG00000006071	*ABCC8*	11p15.1	2.4×10^{-9}
ENSG00000135898	*GPR55*	2q37.1	4.2×10^{-9}
ENSG00000187486	*KCNJ11*	11p15.1	6.6×10^{-9}
ENSG00000109929	*SC5D*	11q23.3-q24.1	7.1×10^{-9}
ENSG00000188211	*NCR3LG1*	11p15.1	7.6×10^{-9}
ENSG00000129159	*KCNC1*	11p15.1	9.3×10^{-9}
-	*LOC101929206*	19p12	1.6×10^{-8}
ENSG00000149403	*GRIK4*	11q23.3	2.2×10^{-8}
ENSG00000113360	*DROSHA*	5p13.3	5.5×10^{-8}
ENSG00000213096	*ZNF254*	19p12	6.4×10^{-8}
ENSG00000082213	*C5orf22*	5p13.3	9.5×10^{-8}
ENSG00000115053	*NCL*	2q37.1	1.1×10^{-7}
ENSG00000133401	*PDZD2*	5p13.3	2.3×10^{-7}
ENSG00000168826	*ZBTB49*	4q16.3	2.6×10^{-7}
ENSG00000135916	*ITM2C*	2q37.1	4.5×10^{-7}
ENSG00000213967	*ZNF726*	19p12	9.1×10^{-7}
ENSG00000168824	*NSG1*	4p16.3	1.6×10^{-6}
ENSG00000198729	*PPP1R14C*	6q25.1	1.8×10^{-6}
ENSG00000145220	*LYAR*	4p16.3	1.9×10^{-6}

Supplementary Table 3. ARHI and hearing loss association

Association between vertigo variants identified in the meta-analysis and ARHI and hearing loss. The definition of the ARHI sample (N_{cases} = 121,934, $N_{controls}$ = 591,699) is described elsewhere[1]. The hearing loss phenotypes were defined by ICD-10 code H90 and ICD-9 codes 389.0-389.2 (conductive and sensorineural hearing loss; N_{cases} = 22,879, $N_{controls}$ = 926,687) and ICD-10 code H91 and ICD-9 codes 388.0, 388.2, 389.7-389.9 (other hearing loss; N_{cases} = 20,845, $N_{controls}$ = 923,892) in Iceland, the UK, the US, and Finland. Significance threshold is based on the number of tests performed ($P \leq 0.05/18 = 2.8 \times 10^{-3}$). *CI* confidence interval.

			**ARHI**		**Conductive and sensorineural hearing loss**		**Other hearing loss**	
**SNP**	**Position (hg38)**	**Gene**	***P***	**OR (95% CI)**	***P***	**OR (95% CI)**	***P***	**OR (95% CI)**
rs612969	chr11:121118626	*TECTA*	9.9×10^{-5}	1.02 (1.01, 1.03)	0.32	0.99 (0.97, 1.01)	0.69	1.00 (0.97, 1.02)
rs10862089	chr12:80305695	*OTOGL*	0.0040	1.03 (1.01, 1.05)	0.77	1.01 (0.97, 1.04)	0.28	0.99 (0.96, 1.01)
rs6753393	chr2:231197253	*ARMC9*	0.35	1.01 (0.99, 1.02)	0.97	1.00 (0.98, 1.02)	0.70	1.00 (0.98, 1.03)
rs2272744	chr4:4248406	*OTOP1*	0.65	1.00 0.99, 1.01)	0.22	0.99 (0.97, 1.01)	0.87	1.00 (0.98, 1.02)
rs7130190	chr11:17558628	*OTOG*	0.76	1.00 (0.99, 1.02)	0.35	1.01 (0.99, 1.04)	0.21	1.02 (0.99, 1.06)
rs428549	chr19:23359488	*ZNF91*	0.78	1.00 (0.99, 1.01)	0.88	1.00 (0.98, 1.02)	0.43	0.99 (0.97, 1.01)

Supplementary Table 4. Translation of codes in the UK dataset

Translation of general practice codes Read Codes Version 2 and Read Codes Clinical Terms Version 3 to ICD-10 codes.

General practice code	ICD-10 code	Description
F56.	H81	Vertiginous syndromes and other disorders of vestibular system
F560.	H81.0	Menière’s disease, endolymphatic hydrops, Lermoyez‘s syndrome
F5600	H81.0	Unspecified Menière’s disease
F5601	H81.0	Active cochleovestibular Menière’s disease
F5602	H81.0	Active cochlear Menière’s disease
F5603	H81.0	Active vestibular Menière’s disease
F5604	H81.0	Inactive Menière’s disease
F560z	H81.0	Menière’s disease NOS
F561.	H81.3	Other and unspecified peripheral vertigo
F5610	H81.3	Unspecified peripheral vertigo
F5611	H81.1	Benign paroxysmal positional vertigo or nystagmus
F5612	H81.2	Acute vestibular neuritis
F5613	H81.2	Recurrent vestibular neuritis
F5614	H81.3	Otogenic vertigo, aural vertigo
F5615	H81.1	Benign paroxysmal positional vertigo
F561z	H81.3	Other peripheral vertigo NOS
F562z	H81.4	Vertigo of central origin
F5620	H81.4	Central positional nystagmus
F5621	H81.4	Malignant positional vertigo
F562z	H81.4	Vertigo of central origin NOS

Supplementary References

1. Ivarsdottir, E. V. *et al.* The genetic architecture of age-related hearing impairment revealed by genome-wide association analysis. *Commun. Biol. 2021 41* **4**, 1–13 (2021).
