# Supplementary material for: A genome-wide meta-analysis uncovers six sequence variants conferring risk of vertigo
Source: Commun Biol. 2021 Oct 7;4:1148. doi: 10.1038/s42003-021-02673-2 (PMC8497462; doi:10.1038/s42003-021-02673-2)
Supplement: Supplementary file 3 — Description of Additional Supplementary Files [file 42003_2021_2673_MOESM3_ESM.pdf]

Description of Additional Supplementary Files

File name: Supplementary Data.

Description: Source data and big supplementary tables.
