# Supplementary material for: A genome-wide meta-analysis uncovers six sequence variants conferring risk of vertigo
Source: Commun Biol. 2021 Oct 7;4:1148. doi: 10.1038/s42003-021-02673-2 (PMC8497462; doi:10.1038/s42003-021-02673-2)
Supplement: Supplementary file 5 — Reporting Summary [file 42003_2021_2673_MOESM5_ESM.pdf]

nature research

Corresponding author(s): Astros Thorarensen Skuladottir, Kari Stefansson

Last updated by author(s): Aug 17, 2021

Reporting Summary

Nature Research wishes to improve the reproducibility of the work that we publish. This form provides structure for consistency and transparency in reporting. For further information on Nature Research policies, see our Editorial Policies and the Editorial Policy Checklist.

Statistics

For all statistical analyses, confirm that the following items are present in the figure legend, table legend, main text, or Methods section.

n/a	Confirmed	
☐	☒	The exact sample size (n) for each experimental group/condition, given as a discrete number and unit of measurement
☒	☐	A statement on whether measurements were taken from distinct samples or whether the same sample was measured repeatedly
☐	☒	The statistical test(s) used AND whether they are one- or two-sided<br>*Only common tests should be described solely by name; describe more complex techniques in the Methods section.*
☐	☒	A description of all covariates tested
☐	☒	A description of any assumptions or corrections, such as tests of normality and adjustment for multiple comparisons
☐	☒	A full description of the statistical parameters including central tendency (e.g. means) or other basic estimates (e.g. regression coefficient) AND variation (e.g. standard deviation) or associated estimates of uncertainty (e.g. confidence intervals)
☐	☒	For null hypothesis testing, the test statistic (e.g. F, t, r) with confidence intervals, effect sizes, degrees of freedom and P value noted<br>*Give P values as exact values whenever suitable.*
☒	☐	For Bayesian analysis, information on the choice of priors and Markov chain Monte Carlo settings
☒	☐	For hierarchical and complex designs, identification of the appropriate level for tests and full reporting of outcomes
☒	☐	Estimates of effect sizes (e.g. Cohen's d, Pearson's r), indicating how they were calculated

Our web collection on statistics for biologists contains articles on many of the points above.

Software and code

Policy information about availability of computer code

Data collection

The FinnGen database consists of samples collected from the Finnish biobanks and phenotype data collected at the national health registers. The summary statistics for vertigo was imported on November 30th, 2020 from a source available to consortium partners (version 4; http://r4.finngen.fi).

Data analysis

GraphTyper2 is a software which uses pangenome graphs to genotype structural variants and small variants using short-reads. GraphTyper is available at https://github.com/DecodeGenetics/graphtyper (v2.0-beta, GNU GPLv3 license). Svimmer, the structural variant merging software is available at https://github.com/DecodeGenetics/svimmer (v0.1, GNU GPLv3 license).

ADMIXTURE is a program used for model-based estimation of ancestry in unrelated individuals. The ADMIXTURE program (v1.2336) is freely available at http://www.genetics.ucla.edu/software.

KING is a software package for relationship inference using high-throughput genotype data that allows the presence of unknown population substructure. KING (v. 2.2.5 --kinship) is available at http://people.virginia.edu/?wc9c/KING.

SMARTPCA is a software package for running eigenanalysis in a LINUX environment. SMARTPCA is available at http://rd.plos.org/david_reich_laboratory.

PLINK is a C/C++ WGAS tool set. With PLINK, large datasets comprising hundreds of thousands of markers genotyped for thousands of individuals can be rapidly manipulated and analyzed in their entirety. PLINK also supports some novel approaches to whole-genome data that take advantage of whole-genome coverage. PLINK (v1.90b6.15) is available at http://pngu.mgh.harvard.edu/purcell/plink/.

UMAP is a manifold learning technique for dimension reduction. UMAP is available at https://github.com/lmcinnes/umap.

SHAPEIT4 is a method that exhibits sub-linear running times with sample size, provides highly accurate haplotypes and allows integrating

external phasing information such as large reference panels of haplotypes, collections of pre-phased variants and long sequencing reads. SHAPEIT4 is available at https://odelaneau.github.io/shapeit4/.

PLINK 2.0 offers dramatic improvements in performance and compatibility from PLINK. Users without access to high-end computing resources can perform several essential analyses of the feature-rich and very large genetic datasets coming into use. PLINK 2.0 is available at https://www.cog-genomics.org/plink2/ (https://github.com/chrchang/plink-ng).

qqman is an R package for visualizing GWAS results in the form of Q-Q and manhattan plots. The source code is available at https://github.com/stephenturner/qqman.

MAGMA v1.08 is a tool for gene and gene-set analysis of GWAS genotype data. MAGMA is available at http://ctglab.nl/software/magma.

GTEx is an ongoing project to build a comprehensive public resource to study tissue-specific gene expression and regulation. The GTEx Portal website is https://www.gtexportal.org/home/.

For manuscripts utilizing custom algorithms or software that are central to the research but not yet described in published literature, software must be made available to editors and reviewers. We strongly encourage code deposition in a community repository (e.g. GitHub). See the Nature Research guidelines for submitting code & software for further information.

Data

Policy information about availability of data

All manuscripts must include a data availability statement. This statement should provide the following information, where applicable:

- Accession codes, unique identifiers, or web links for publicly available datasets
- A list of figures that have associated raw data
- A description of any restrictions on data availability

The GWAS results from this study are deposited at https://www.decode.com/summarydata/.

GWAS summary statistics from Finland are restricted to researchers representing the consortium partners and were downloaded from https://r4.finngen.fi/pheno/H8_VERTIGO, https://r4.finngen.fi/pheno/H8_MENIERE, https://r4.finngen.fi/pheno/H8_BPV, and https://r4.finngen.fi/pheno/H8_VESTIBNEUR.

Other data generated or analyzed during this study are included in Supplementary Data.

Field-specific reporting

Please select the one below that is the best fit for your research. If you are not sure, read the appropriate sections before making your selection.

☒ Life sciences ☐ Behavioural & social sciences ☐ Ecological, evolutionary & environmental sciences

For a reference copy of the document with all sections, see nature.com/documents/nr-reporting-summary-flat.pdf

Life sciences study design

All studies must disclose on these points even when the disclosure is negative.

Sample size	The sample size was determined by combining all available subjects that had a vertigo/vestibular disorder diagnosis in the four available datasets.
Data exclusions	We excluded variants with imputation information below 0.8 and MAF below 0.01% for quality reasons.
Replication	Our study was a meta-analysis of available GWAS studies with no direct replication. However, in a subtype analysis, we did not replicate previously published variants associated with vestibular neuritis in our vestibular neuritis meta-analysis and we found evidence of heterogeneity between the effects of the two studies.
Randomization	No randomizations were used in this study as this is a GWAS study.
Blinding	This is an observational study and no blinding was required.

Reporting for specific materials, systems and methods

We require information from authors about some types of materials, experimental systems and methods used in many studies. Here, indicate whether each material, system or method listed is relevant to your study. If you are not sure if a list item applies to your research, read the appropriate section before selecting a response.

Materials & experimental systems

n/a	Involved in the study	
☒	☐	Antibodies
☒	☐	Eukaryotic cell lines
☒	☐	Palaeontology and archaeology
☒	☐	Animals and other organisms
☐	☒	Human research participants
☒	☐	Clinical data
☒	☐	Dual use research of concern

Methods

n/a	Involved in the study	
☒	☐	ChIP-seq
☒	☐	Flow cytometry
☒	☐	MRI-based neuroimaging

Human research participants

Policy information about studies involving human research participants

Population characteristics	The study included individuals diagnosed with vertigo or vestibular disorders in Iceland, the UK, the US, and Finland that have participated in a research program at deCODE genetics, the UK Biobank, HerediGene: Population study, or Finngen.<br>The ratio of female to male was comparable in all four datasets, ~66% were females (mean age at first event ~54) and ~33% were males (mean age at first event ~57). We did not have age at first event for the US dataset.
Recruitment	In Iceland, a large fraction of the population of 360,000 inhabitants has participated in a nationwide research program at deCODE genetics. Participants in this current study were identified in the Registry of Primary Health Care Contacts and the Registry of Contacts with Medical Specialists in Private Practice, which covers the entire population for the recorded period, and recruited into the study based on available data in the nationwide research program.<br>The UK Biobank study is a large cohort study of ~500,000 participants in the age range of 40-69. Participants in this study were identified from General Practice clinical recent records and from the UK hospital diagnoses.<br>The US participants in the HerediGene: Population study are volunteers over the age of 18. US participants were identified using medical records.<br>The Finngen study is a partnership between Finnish universities, biobanks, hospital districts, and several international pharmaceutical companies and is open to every Finn to participate.<br><br>Using this approach, any selection bias is unlikely and thus, it is unlikely that the recruitment method has any impact on the results.
Ethics oversight	All Icelandic data were collected by studies approved by the National Bioethics Committee (NBC; VSN-19-158; VSNb2019090003/03.01) following review by the Icelandic Data Protection Authority.<br><br>The UK Biobank data were obtained under application number 24898. The North West Research Ethics Committee reviewed and approved UK Biobank's scientific protocol and operational procedures (REC Reference Number: 06/MRE08/65).<br><br>The US data from HerediGene: Population study was approved by The Intermountain Healthcare Institutional Review Board.<br><br>The Finngen data was approved by The Coordinating Ethics Committee of the Helsinki and Uusimaa Hospital Distric.

Note that full information on the approval of the study protocol must also be provided in the manuscript.
